# Surfactant Secretion in LRRK2 Knock-Out Rats: Changes in Lamellar Body Morphology and Rate of Exocytosis

**DOI:** 10.1371/journal.pone.0084926

**Published:** 2014-01-21

**Authors:** Pika Miklavc, Konstantin Ehinger, Kristin E. Thompson, Nina Hobi, Derya R. Shimshek, Manfred Frick

**Affiliations:** 1 Institute of General Physiology, University of Ulm, Ulm, Germany; 2 Department of Physiology and Medical Physics, Innsbruck Medical University, Innsbruck, Austria; 3 Department of Autoimmunity/Transplantation/Inflammation-Neuroscience, Novartis Institutes for BioMedical Research, Basel, Switzerland; University of Padova, Italy

## Abstract

Leucine-rich repeat kinase 2 (LRRK2) is known to play a role in the pathogenesis of various diseases including Parkinson disease, morbus Crohn, leprosy and cancer. LRRK2 is suggested to be involved in a number of cell biological processes such as vesicular trafficking, transcription, autophagy and lysosomal pathways. Recent histological studies of lungs of LRRK2 knock-out (LRRK2 -/-) mice revealed significantly enlarged lamellar bodies (LBs) in alveolar type II (ATII) epithelial cells. LBs are large, lysosome-related storage organelles for pulmonary surfactant, which is released into the alveolar lumen upon LB exocytosis. In this study we used high-resolution, subcellular live-cell imaging assays to investigate whether similar morphological changes can be observed in primary ATII cells from LRRK2 -/- rats and whether such changes result in altered LB exocytosis. Similarly to the report in mice, ATII cells from LRRK2 -/- rats contained significantly enlarged LBs resulting in a >50% increase in LB volume. Stimulation of ATII cells with ATP elicited LB exocytosis in a significantly increased proportion of cells from LRRK2 -/- animals. LRRK2 -/- cells also displayed increased intracellular Ca^2+^ release upon ATP treatment and significant triggering of LB exocytosis. These findings are in line with the strong Ca^2+^-dependence of LB fusion activity and suggest that LRRK2 -/- affects exocytic response in ATII cells via modulating intracellular Ca^2+^ signaling. Post-fusion regulation of surfactant secretion was unaltered. Actin coating of fused vesicles and subsequent vesicle compression to promote surfactant expulsion were comparable in cells from LRRK2 -/- and wt animals. Surprisingly, surfactant (phospholipid) release from LRRK2 -/- cells was reduced following stimulation of LB exocytosis possibly due to impaired LB maturation and surfactant loading of LBs. In summary our results suggest that LRRK2 -/- affects LB size, modulates intracellular Ca^2+^ signaling and promotes LB exocytosis upon stimulation of ATII cells with ATP.

## Introduction

LRRK2 is a ∼280 kDa protein with two enzymatic domains (Ras of complex GTPase domain and kinase domain) and several protein-protein interaction domains such as an amino terminal leucine-rich repeat domain and a carboxy terminal WD40 domain [1], [2]. LRRK2 and mutations thereof have been found to play a role in the pathogenesis of various diseases. Mutations in LRRK2 are associated with the familial form of Parkinson disease [3]–[7] but were also linked to inflammatory bowel disease [8], leprosy [9], and cancer [10]. Recent findings suggested an important role for LRRK2 in immune-response, which may explain the wide variety of diseases associated with LRRK2 mutations [11]. LRRK2 is expressed in the cells of the immune system and was suggested to be involved in monocyte maturation [12], [13]. It is also involved in regulation of microglial inflammatory responses which may be associated with late-onset Parkinson disease [14], [15].

Despite the importance of LRRK2 for the pathogenesis in various diseases little is known about the cellular function of LRRK2. LRRK2 has been implicated in many different signaling pathways such as membrane trafficking [16], apoptosis [17], cytoskeletal remodeling [18], and transcriptional regulation [19]. LRRK2 was also described to modulate synaptic transmission [20]. Silencing of LRRK2 in cortical neurons resulted in altered availability of synaptic vesicles, increased vesicle fusion rate and impaired compensatory endocytosis [21], [22]. LRRK2 was also suggested to play a role in lysosomal trafficking [23]–[25]. Gain-of-function mutation in the LRRK2 kinase domain caused spherical inclusions reminiscent of swollen lysosomes in axons of cultured neurons [26]. In Drosophila, LRRK2 was shown to negatively regulate perinuclear localization of lysosomes [27] and in human brain LRRK2 localizes to vesicles in the lysosomal pathway [28]. A recent study found that LRRK2 -/- mice have an increased number and average size of secondary lysosomes in kidney proximal tubulus cells and LBs in ATII cells in the lung [29]. LBs are lysosome-derived secretory vesicles that store lung surfactant. Upon stimulation surfactant is secreted via exocytosis of LBs. Surfactant consists of lipids and specialized proteins and is secreted into the alveolar lining fluid in order to reduce surface tension of the lungs [30]–[32].

During LB exocytosis a sequence of highly regulated steps leads to fusion of exocytic vesicles with the plasma membrane, subsequent opening of a fusion pore and finally content release. Several intracellular signalling cascades stimulate LB fusion with the plasma membrane during the exocytic pre-fusion phase [30], [33], with changes in the intracellular Ca^2+^ concentration ([Ca^2+^]_c_) being at center stage [34]. Opening of the fusion pore in ATII cells is preceded by lipid mixing of plasma membrane and LB limiting membrane – the hemifusion [35]. Due to its tight packing and the highly lipophilic nature surfactant does not readily diffuse out of fused LBs following opening of the fusion pore. At least two additional mechanisms are essential to promote secretion during the post-fusion phase. First, the fusion pore acts as a mechanical barrier for the release [36] and has to widen sufficiently – a process modulated by [Ca^2+^]_c_ [37]. Exocytosis of LBs results in localized Ca^2+^ influx via vesicular P2X_4_ receptors which promotes fusion pore expansion and subsequently facilitates surfactant release [38], [39]. Second, fused LBs acquire an actin coat which compresses the vesicle and thereby actively extrudes poorly soluble surfactant [40], [41].

Within this study we investigated whether morphological changes of LBs observed in LRRK2 -/- mice are also present in LRRK2 -/- rats and whether they affect LB exocytosis and surfactant secretion. We measured single LB fusion events with the plasma membrane using high resolution, real-time fluorescent microscopy. ATII cells are particularly suitable for analysing single exocytotic events due to large size of secretory vesicles and slow, sequential vesicle fusion with plasma membrane. LB exocytosis has been intensively investigated in the past decade and there are several established microscopy methods for detection of single vesicle fusion events [37], [41]–[43]. Similar to the previously reported increase in LB size in LRRK2 -/- mice [29], we found that LB size was also significantly increased in primary ATII cells from LRRK2 -/- rats compared to LB size from wild-type (wt) animals. Other morphological properties of lamellar bodies did not seem to differ from wt animals. We also found that exocytotic activity upon stimulation with ATP was significantly increased in ATII cells from LRRK2 -/- animals. This is likely due to an increased Ca^2+^ release from intracellular stores upon stimulation with ATP, which promotes triggering of LB exocytosis. However, using a biochemical approach to quantify surfactant secretion we found that surfactant release to the extracellular space was decreased after stimulation. This was not due to failure of actin coat formation and active extrusion of vesicle contents during the exocytic post fusion phase. In summary our results suggest that LRRK2 -/- affects LB size, modulates intracellular Ca^2+^ signalling and promotes LB exocytosis upon stimulation of ATII cells with ATP.

## Materials and Methods

### Ethics Statement

Rats were maintained at the central animal facility of the Ulm University according to institutional guidelines for ethical care of animals. All experiments in this study were approved by Regierungspräsidium Tübingen, grant Nr. 833.

### Rats

LRRK2 -/- rats were purchased from SAGE Labs (Long Evans, nomenclature: LEH-Lrrk2^tm1sage^, product number: TGRL4620) as well as age- and gender-matched wildtypes (Long-Evans). The age of animals ranged between 5 and 9 weeks.

### Alveolar type II cell isolation

Alveolar type II cells were isolated from wt and LRRK2 -/- rats according to the procedure of Dobbs et al. [44] with minor modifications as recently described [41]. In short, rats were anesthetized with ketamin (10%) and xylazil (2%), and injected with heparin (400 IU/kg). Lungs were perfused, removed, washed and incubated with elastase and trypsin at 37°C two times for 15 minutes. Afterwards, the lungs were immersed in DNAse containing solution and sliced with scissors into bits of about 1 mm^3^. Enzyme reaction was blocked by incubation with FCS at 37°C for 2 min. The digested tissue was filtered 3 times through gauze and nylon meshes and the final filtrate was centrifuged for 8 min at 130×g. After resuspension in DMEM medium, the cells were incubated on IgG coated plastic dishes at 37°C for 15 min. Non-adherent cells were centrifuged for 8 min at 130×g, suspended in MucilAir medium (Epithelix, Geneva, Switzerland), seeded on chamber slides (Ibidi, Martinsried, Germany), and used for experiments for up to 48 h after seeding.

### Immunofluorescence

Cells were washed twice in ice-cold DPBS (pH 7.4, Biochrom, Berlin, Germany) fixed for 20 min in 4% paraformaldehyde (Sigma, Schnelldorf, Germany) in DPBS and permeabilised for 10 min with 0.2% saponin and 10% FBS (Thermo Scientific, Bonn, Germany) in DPBS. Cells were subsequently stained with primary (1∶300) and secondary (1∶400) antibodies in PBS, 0.2% saponin and 10% FBS for 30 min. Images were taken on an inverted confocal microscope (Leica TCS SP5, Leica, Germany) using a 63× lens (Leica HCX PL APO lambda blue 63.0×1.40 OIL UV). Images for the blue (DAPI), green (AlexaFluor 488), red (AlexaFluor 568) and far red (AlexaFluor 633) channel were taken in sequential mode using appropriate excitation and emission settings. All used primary antibodies were from Abcam (Cambridge, UK) apart from antibodies against surfactant proteins B and C which were a gift from T. Haller (Innsbruck Medical University, Austria). AlexaFluor phalloidin 568 and all conjugated secondary antibodies were from Invitrogen.

### Live-cell microscopy

The fura-2/FM1-43 assay for combined measurement of changes in the [Ca^2+^]_c_ and detection of individual LB fusion events was performed as described previously [43]. In brief, primary ATII cells were loaded with 3 µM fura-2 AM for 20 min in MucilAir, washed twice in bath solution (in mM: 140 NaCl, 5 KCl, 1 MgCl_2_, 2 CaCl_2_, 5 glucose, 10 Hepes; pH 7.4) and kept in bath solution with 0.5 – 1 μM of FM 1–43. To efficiently induce LB exocytosis ATII cells were treated with various known and potent agonists for surfactant secretion: ATP (100 μM), PMA (300 nM), a combination of ATP and PMA, or ionomycin (1 μM) (all from Sigma, Schnelldorf, Germany). The combined application of ATP and PMA was used because the previous report showed that the combined application can further increase LB fusion probability [42] The experiments were performed on an iMic digital microscope (Till Photonics, Germany) and on a Cell Observer inverse microscope (Zeiss, Germany). Cells were illuminated for 50 ms at a rate of 0.3–0.5 Hz at each excitation wavelength (340 and 380 nm for Fura-2; 480 nm for FM 1–43). A 495 nm (Observer) and a 520 nm dichroic mirror (iMic) were used to deflect excitation light. In this setting, channel crosstalk between the FM 1–43 fluorescence and the fura-2 ratio would lead to small under-estimations of [Ca^2+^]_c_ as described in detail earlier [43]. Images were acquired using MetaFluor (Molecular Devices, Ismaning, Germany) or iMic Online Analysis (Till Photonics, Germany). Images were analysed using MetaFluor Analyst (Molecular Devices, Ismaning, Germany) or iMic Offline analysis software (Till Photonics, Germany). FM1-43 intensities were analysed in a region encircling the fusing LBs. Fura-2 fluorescence was determined in a region of interest surrounding individual cells.

For live-cell actin-coating experiments we transfected ATII cells with actin-GFP using a previously described adenoviral transfection system [40], [41]. Before experiments cells were incubated with LysoTracker Red (LTR, LifeTechnologies, Germany; 100 nM, 10 min) to detect LB fusions. LysoTracker dyes accumulate in LBs and rapidly diffuse out of the vesicle after fusion. Experiments were performed on the iMic digital microscope (Till Photonics, Germany) with a 488 nm excitation filter for actin GFP and 568 nm excitation filter for LTR. We analysed the fraction of LBs that were coated with actin following fusion (as determined by loss of LTR fluorescence) and the fraction of actin coats that contracted within 30 s and 60 s following actin coat formation.

### Measurement of LB size

LB size was measured in cells stained with either Nile red (200 nM, 5 min; Life Technologies Germany) or metabolically labeled using Bodipy phosphatidylcholine (100 µM, 24 h, Life Technologies Germany). Nile red, an unspecific lipid marker is, due to the high phospholipid content of surfactant, predominately trapped within LBs [35] whereas Bodipy phosphatidylcholine selectively incorporates into surfactant stored in LBs [40], [45], [46]. Images were taken on the Cell Observer inverse microscope and the area of fluorescence was measured for individual vesicles in ImageJ. LB area was used to calculate vesicle diameter.

### Quantitation of phosphatidylcholine release

Isolated ATII cells were seeded into 6-well plates (∼0.5×10^6^/well). At day 2 after isolation, cells were washed 3 times in bath solution and stimulated with either ATP (100 µM) or PMA (300 nM). The total volume in each well following stimulation was 0.5 ml. Cells were incubated for 15 min on a horizontal shaker (120 rpm, 37°C) before supernatants were collected and analyzed for dipalmitoylphosphatidylcholine (DPPC) content in triplicate for each sample using coupled enzymatic reactions as described previously [47].

### Calculation of cytoplasmic Ca^2+^ concentration

We estimated the intracellular free Ca^2+^ concentration in isolated ATII cells as described in detail previously [48]–[50] using the equation [Ca^2+^]_c_ = K_d_×[(R−R_min_)/(R_max_−R)]×(S_f2_/S_b2_).

ATII cells were seeded in perfusion chambers (Ibidi, Martinsried, Germany) and stained with fura-2 AM (3 µM for 20 min). Ratio (R) was calculated from 340/380 nm excitation intensities in unstimulated cells in normal experimental bath solution. R_min_ was measured after Ca^2+^ ionophore ionomycin (20 µM) was added to a Ca^2+^ free perfusion solution (in mM: 135 NaCl, 5 KCl, 1 MgCl_2_, 5 glucose, 5 Hepes, 5 EGTA). R_max_ was measured after ionomycin (20 µM) addition to Ca^2+^-containing perfusion solution (in mM: 134 NaCl, 5 KCl, 1 MgCl_2_, 5 CaCl_2_, 5 glucose, 5 Hepes). The proportionality coefficient S_f2_ was measured as the maximal 380 nm fluorescence intensity after ionomycin addition to Ca^2+^-free solution. The second proportionality coefficient, S_b2_, was measured as the minimal 380 nm fluorescence intensity after ionomycin addition to Ca^2+^-containing solution. We corrected both coefficients for the background fluorescence which was measured after MnCl_2_ quench (in mM: 140 NaCl, 5 Hepes, 5 MnCl_2_) at the end of the experiment. We used 224 nM as a K_d_ value for fura-2 [48].

### Calculation of intravesicular pH in LBs

ATII cells were incubated with 100 nM LysoSensor Yellow/Blue (Invitrogen) at 37°C for 30 min. Cells were washed with experimental bath solution and imaged on the Zeiss Observer microscope (dichroic filter 400 nm, emission filter 490–530 nm) and 40× fluar oil objective using Metafluor software. After background subtraction, LB intensities at 340 and 380 nm excitation were measured in ImageJ and the 340/380 nm ratio was used for pH calculation.

Calibration of the ratio to pH relation was done as described previously [51] by a free solution calibration. Bath solution with 1 µM LysoSensor was adjusted to pH values from 3 to 8 and imaged as described. The calibration curve could be fitted well with pH = c−log_10_((a−b)/(ratio−b)−1), where a, b and c are constants.

### Statistical analysis

We used Microsoft Excel and GraphPad Prism (GraphPad Software Inc., San Diego, USA) for statistical analysis. Unless stated otherwise data are expressed as mean +/− SEM. n denotes true biological replicates (i.e. animals) and non-paired student's t-test was used to assess the statistical differences between wt and LRRK2 -/- cell populations. Unless stated otherwise the number of experiments performed/cells analysed in each biological replicate was kept constant to account for correct weighting of individual biological replicates when generating means. Due to the low number of fusions/cell (on average 1–2 per responding cell) and within individual experiments (6–8 responding cells) fusion delay histograms were derived from pooled data from 3 to 6 animals. A non-parametric Mann-Whitney test was used to compare the medians of fusion delays after stimulation.

## Results

### Morphological properties of LBs from LRRK2 -/- cells

**Figure 1:**
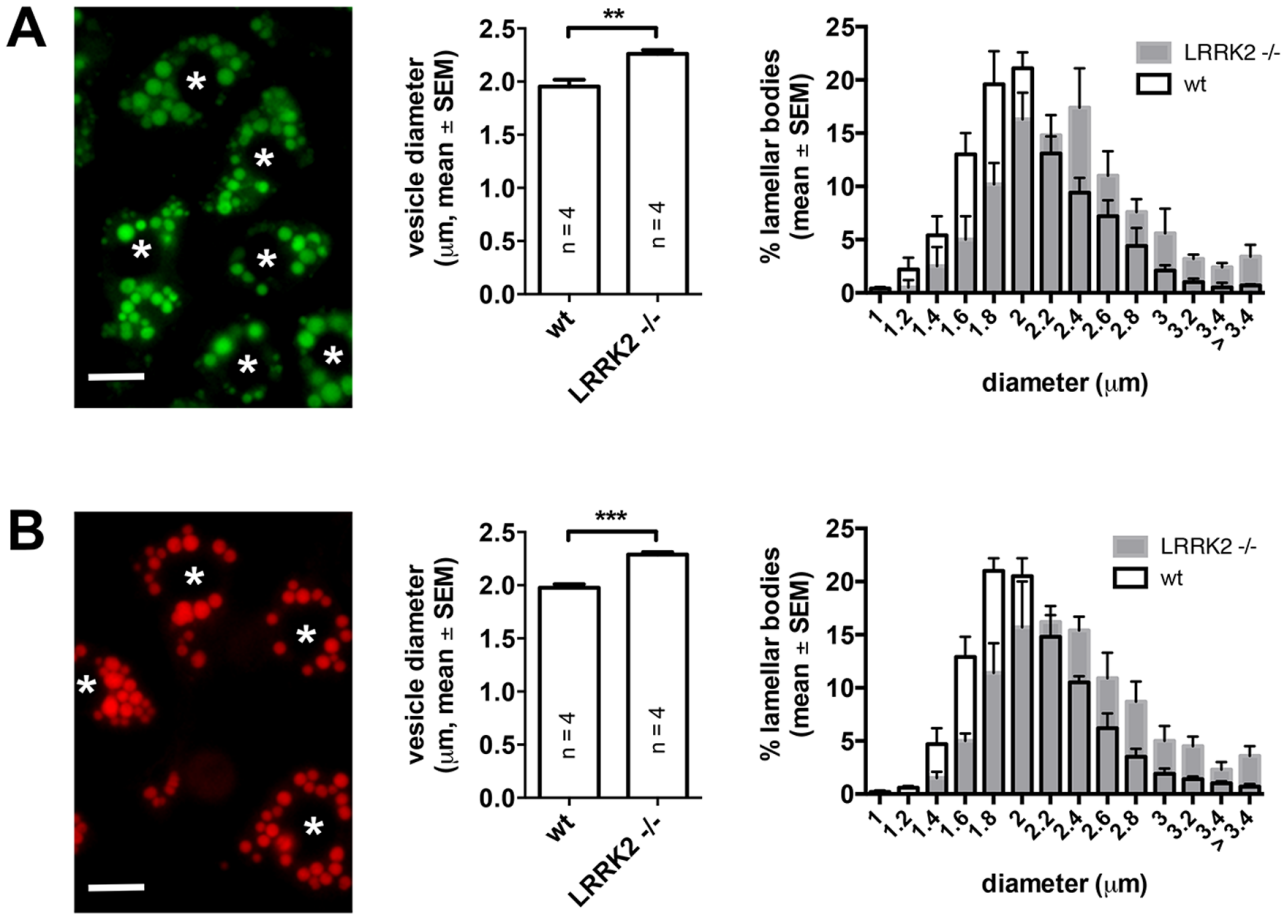
LBs are significantly enlarged in LRRK2 -/- rats. (A) LBs (green) in cells that were metabolically labeled with Bodipy phosphatidylcholine (left, asterisks indicate nuclei, scale bar  = 10 µm). LB size was significantly (p<0.006) increased in cells from LRRK2 -/- animals (diameter: 2.26 µm±0.04, n = 4 animals) compared to LBs in cells from wt animals (diameter: 1.95 µm±0.07, n = 4 animals) (middle). Size distribution diagram for LB sizes derived from wt and LRRK2 -/- animals (right, n = 4 animals). (B) LBs (red) in cells that were stained with lipophilic dye Nile red (left, asterisks indicate nuclei, scale bar  = 10 µm). LB size was significantly (p<0.0003) increased in cells from LRRK2 -/- animals (diameter: 2.29 µm±0.03, n = 4 animals) compared to LBs in cells from wt animals (diameter: 1.98 µm±0.03, n = 4 animals) (middle). Size distribution diagram for LB sizes derived from wt and LRRK2 -/- animals (right, n = 4 animals). In all experiments 25 cells were analyzed in each animal corresponding to approx. 350 – 450 LBs per animal. Images were taken from LRRK2 –/- cells.

The previous histological study of the lungs from LRRK2 -/- mice showed enlarged size and number of LBs in ATII cells [29]. In this study we used fluorescence microscopy and immunocytochemistry to analyse LB size, expression and localization of LB markers as well as functional parameters of LBs in ATII cells isolated from LRRK2 -/- and wt rats. Analyzing the size of LBs in cells that were metabolically labeled with Bodipy phosphatidylcholine revealed that LB size was significantly (p<0.006) increased in cells from LRRK2 -/- animals (diameter: 2.26 µm±0.04, n = 4) compared to LBs in cells from wt animals (diameter: 1.95 µm±0.07, n = 4) (Figure 1A). Similar results were obtained in cells when LBs were stained with Nile red, an unspecific lipid marker that is predominately trapped within LBs [35]. LB size was again significantly (p<0.0003) increased in cells from LRRK2 -/- animals (diameter: 2.29 µm±0.03, n = 4) compared to LBs in cells from wt animals (diameter: 1.98 µm±0.03, n = 4) (Figure 1B). These changes in diameter correlate to a more than 50% increase in LB volume. In all experiments 20 cells were analyzed in each animal corresponding to approx. 350–450 LBs per animal.

**Figure 2:**
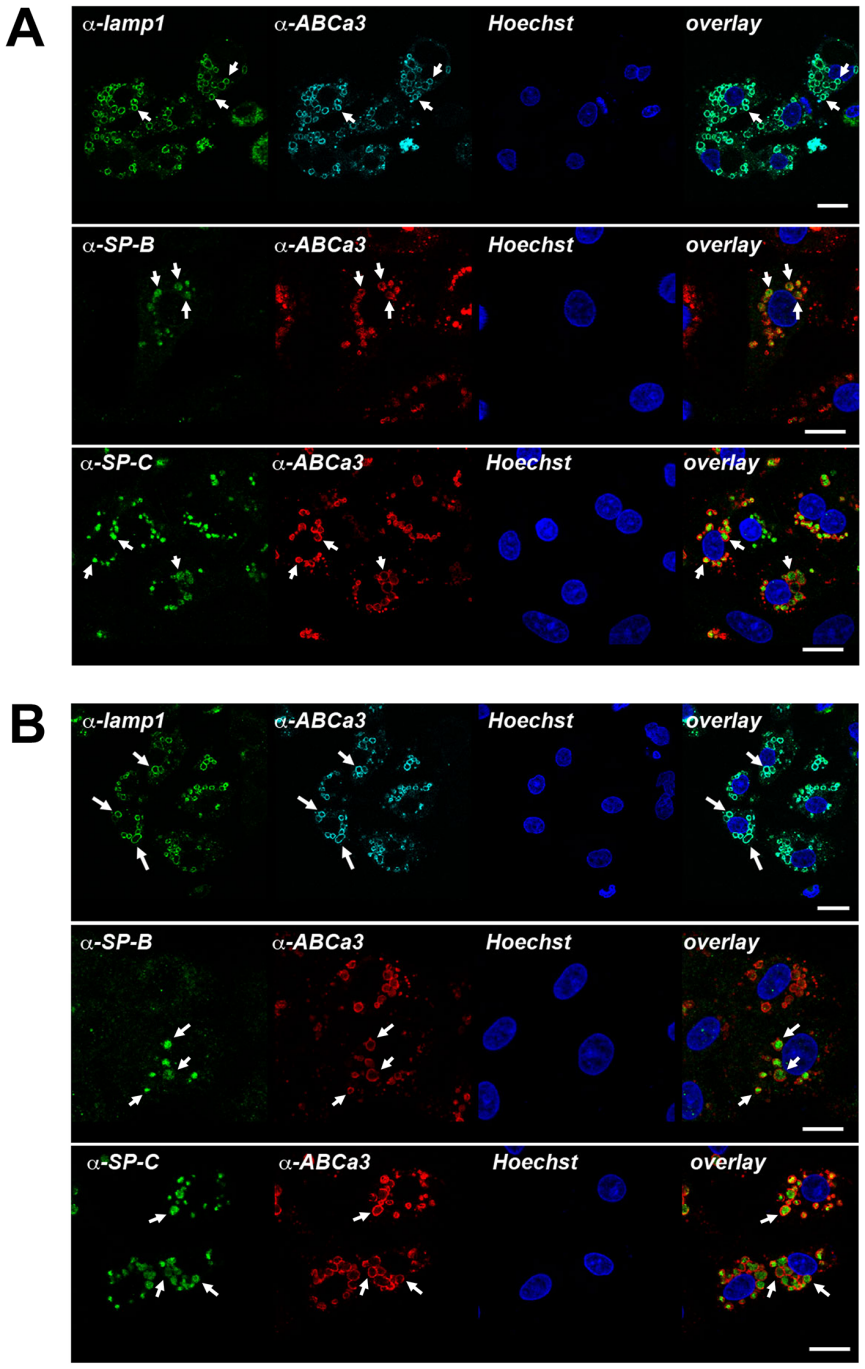
Expression and localization of LB markers are not affected by LRRK2 -/-. Immunocytochemical localization of Lamp1, ABCa3, and surfactant proteins SP-B and SP-C in ATII isolated from LRRK2 -/- rats (A) and ATII cells isolated from wt rats (B). Lamp1 and ABCa3 localized to LB limiting membrane, whereas SP-B and SP-C are inside the vesicle together with the lipid component of surfactant. No obvious differences in localization of these markers in cells isolated from KO and wt rats were observed. Arrows indicate individual LBs in type II cells. Scale bar = 10 µM

Expression and localization of LB markers were not changed. LB membrane marker proteins Lamp1 and ABCa3 transporter were localized to the LB membrane in LRRK2 -/- as well as in wt cells (Figure 2A&B) [38]. Surfactant proteins B and C, which are stored inside LBs together with lipids and function in proper spreading of surfactant [52], were also present in both LRRK2 -/- and wt cells, with no obvious differences in their distribution and abundance (Figure 2A&B).

To further investigate whether the observed increase in LB size is linked to changes in lamellar body homeostasis we also performed experiments to assess the pH in LBs from wt and LRRK2 -/- cells using the dye LysoSensor. No significant difference (p = 0.45) between the vesicular pH in LBs from wt animals (5.12+/−0.05, n = 4) and LBs from LRRK2 -/- animals (5.18+/−0.06, n = 4) could be observed. Approx. 150 to 250 LBs were analyzed per animal.

### LB fusion kinetics and changes in [Ca^2+^]_c_ in LRRK2 -/- cells

**Figure 3:**
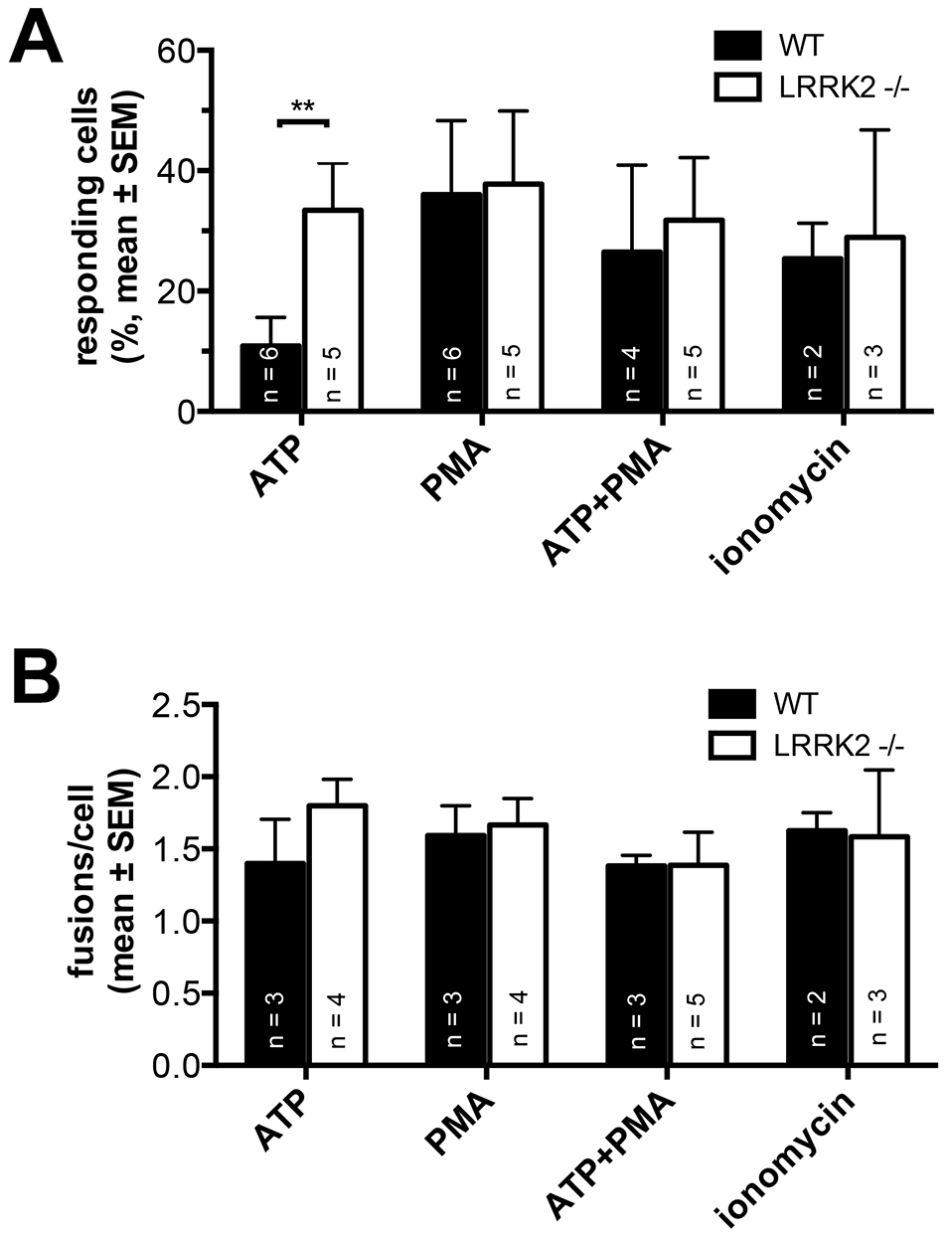
ATP stimulation results in an increased fusion response in LRRK2 -/- cells. Response is expressed as the fraction of cells with at least 1 fusion within 10(A), and as a number of fusions/cell in responding cells (B). n denotes number of animals. Equal numbers of experiments were conducted in each animal.

In initial experiments we tested whether the morphological changes observed in cells from LRRK2 -/- rats affect kinetics of LB exocytosis. Therefore, we stimulated ATII cells isolated from either wt or LRRK2 -/- rats with various secretagogues stimulating surfactant secretion via different signaling pathways [34], [42]. ATP stimulates LB exocytosis mainly via a transient, IP_3_ mediated calcium release from internal calcium stores resulting in a “triggered” fusion response. PMA on the other hand acts via activation of protein kinase C without affecting intracellular Ca^2+^ concentrations and enables a more prolonged response to stimulation whereas ionomycin acts as an ionophore and causes massive Ca^2+^-entry from the extracellular space [34], [42]. The rationale for these experiments was to investigate potential effects of LRRK2 -/- on LB fusion kinetics following activation of different signalling pathways.We measured fusion response in isolated ATII cells using the lipophylic dye FM1-43. FM1-43 is essentially non-fluorescent in aqueous solutions but yields a bright signal when incorporated into lipid layers. Upon fusion of LBs with the plasma membrane and opening of the fusion pore FM1-43 gains access to the LB lumen, incorporates into the lipophilic vesicle contents (i.e. surfactant) and individual fusion events can hence be readily detected [43]. In initial experiments we determined the percentage of cells that responded to stimulation with at least one fusion event within 10 min after stimulation. When cells where stimulated with either 300 nM PMA, 300 nM PMA plus 100 µM ATP or 1 µM ionomycin no difference between the percentage of responding cells in wt and LRRK2 -/- cells could be observed (Figure 3A). All these treatments cause maximum activation of either the PKC (PMA, PMA plus ATP) or Ca^2+^ (ionomycin) signaling pathways and result in comparable activity responses (Figure 3A). However, when cells were stimulated with 100 µM ATP only, a more physiological stimulus [30], [33], [34], which elicits a transient rise in [Ca^2+^]_c_ due to release of Ca^2+^ from intracellular stores, cells from LRRKO -/- animals had a significantly (p = 0.01) increased fusion response compared to wt cells (33.4±7.9%, n = 5 and 10.9±4.7%, n = 6 percent of cells responded to stimulation, respectively) (Figure 3A). When analyzing the number of LB fusions in responding cells, we did not observe a difference between LRRK2 -/- and wt following stimulation with any secretagogue, yet slightly more fusions were observed in LRRK2 -/- responders following stimulation with ATP (Figure 3B). These data suggested that LRRK2 -/- affects exocytic response in ATII cells via modulation of intracellular Ca^2+^ signaling but not vesicle recruitment to the plasma membrane.

**Figure 4:**
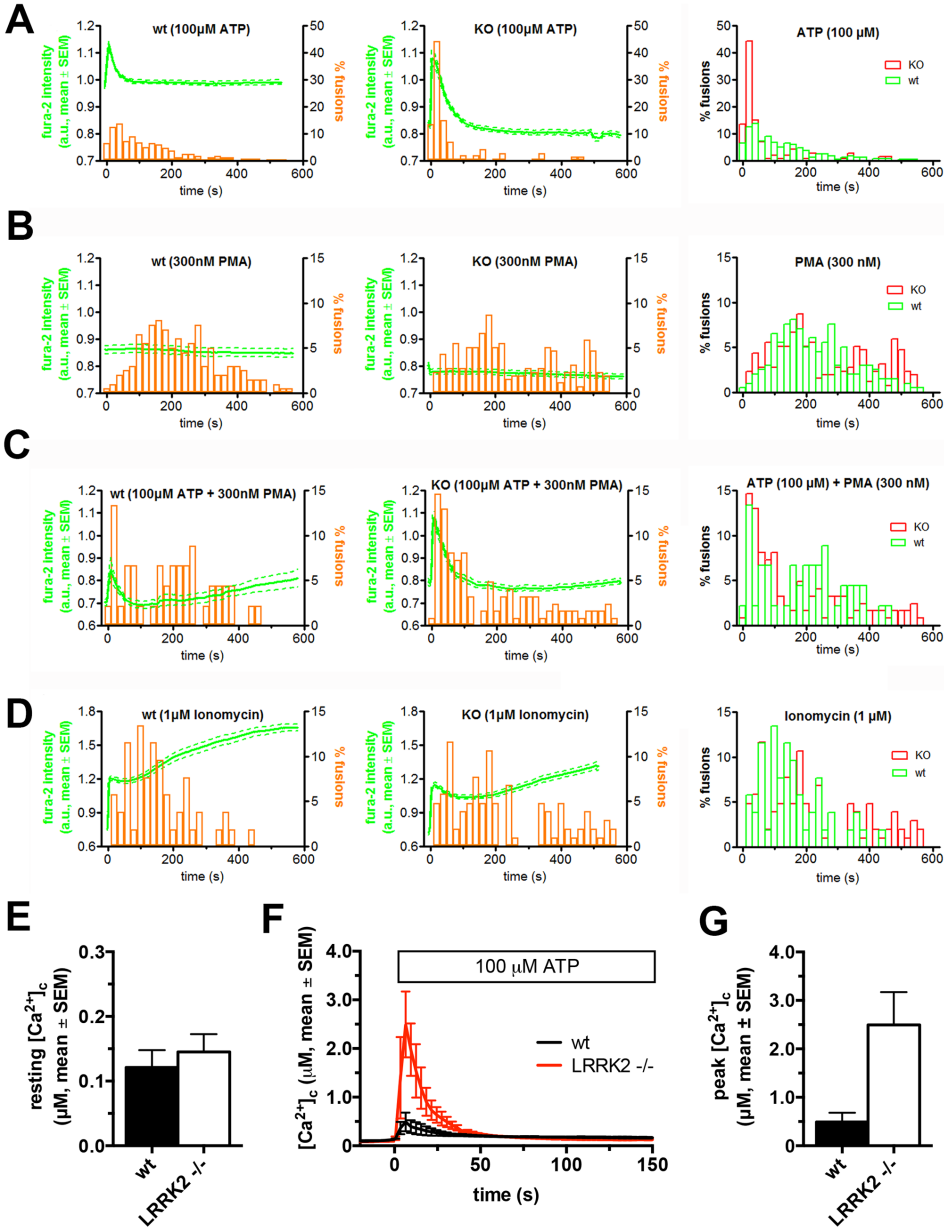
ATP accelerates LB fusion and increases intracellular Ca^2+^-release in LRRK2 -/- cells. LB fusion response time histograms (bars) and fura-2 ratios (lines) in response to stimulation of ATII cells. (A – D) Fusion delay histograms from wt and LRRK2 -/- cells following stimulation with 100 µM ATP (A), 300 nM PMA (B), 100 µM ATP and 300 nM PMA (C) and 1 µM ionomycin (D). Stimulation with ATP resulted in a significant left shift in the fusion delay histograms in LRRK2 -/- cells. Delay histograms were derived from pooled data from 3 to 6 animals per experimental condition and from 45 to 256 individual fusions. Cells were stimulated at t = 0 s. Fura-2 traces were derived from 7 to 116 cells. Panels represent wt cells (left), LRRK2 -/- cells (middle) and overlay of wt and -/- delay histograms (right). (E) Resting [Ca^2+^]_c_ is not significantly enhanced in cells from LRRK2 -/- animals (145.2±27.3 nM, n = 4 in LRRK2 -/- cells and 121.2±26.5 nM, n = 4 in wt cells, respectively, p = 0.55, approx. 15 to 20 cells were analysed for each animal). (F) Changes in [Ca^2+^]_c_ following stimulation with 100 µM ATP (Cells were stimulated at t = 0 s, n = 3 and 2 for LRRK2 -/- and wt cells respectively). (G) Peak [Ca^2+^]_c_ following stimulation with 100 µM ATP in LRRK2 -/- cells (n = 3) compared to wt cells (n = 2).

Based on these findings we further investigated the possible impact of LRRK2 deletion on calcium-stimulated LB fusion activity in ATII cells. We simultaneously analysed changes in [Ca^2+^]_c_ using fura-2 while monitoring individual LB fusion events (Figure 4). Overall [Ca^2+^]_c_ kinetics were comparable between LRRK2 -/- and wt cells following various modes of stimulation (Figure 4A-D). However, in addition to increasing the number of responding cells stimulation with 100 µM ATP also resulted in a significant left shift in the fusion delay histograms (p<0.0001, median delay was 102 s and 37 s for wt and LRRK2 -/- respectively), indicating a pronounced “triggering” of the onset of LB fusions (Figure 4A right). Fusion kinetics in cells stimulated with 100 µM ATP and 300 nM PMA, 300 nM PMA or 1 µM ionomycin were not significantly different in LRRK2 -/- and wt cells (Figure 4B–D, p = 0.17, 0.21, and 0.10, respectively).

LB fusion activity is intimately linked to [Ca^2+^]_c_ [34], [42], [49]. Fusion activity strictly depends on reaching the threshold [Ca^2+^]_c_ of approx. 320 nM and fusion kinetics correlate with [Ca^2+^]_c_ kinetics [42], [49]. We therefore tested whether LRRK2 -/- affects absolute [Ca^2+^]_c_ and whether such an effect could account for the prominent effect on LB fusion response and LB fusion kinetics following stimulation with ATP. Resting [Ca^2+^]_c_ was not significantly enhanced in cells from LRRK2 -/- animals (145.2±27.3 nM, n = 4 in LRRK2 -/- cells and 121.2±26.5 nM, n = 4 in wt cells, respectively, p = 0.55, approx. 15 to 20 cells were analysed for each animal)(Figure 4E). However, the transient rise in [Ca^2+^]_c_ upon stimulation with 100 µM ATP resulting from intracellular release of Ca^2+^ was increased in cells from LRRK2 -/- animals (Figure 4F). Mean peak [Ca^2+^]_c_ reached 2.49±0.68 µM (n = 3) in LRRK2 -/- cells and 0.49±0.19 µM (n = 2) in wt cells, respectively (p = 0.08) (Figure 4G).

### Surfactant secretion assay

**Figure 5:**
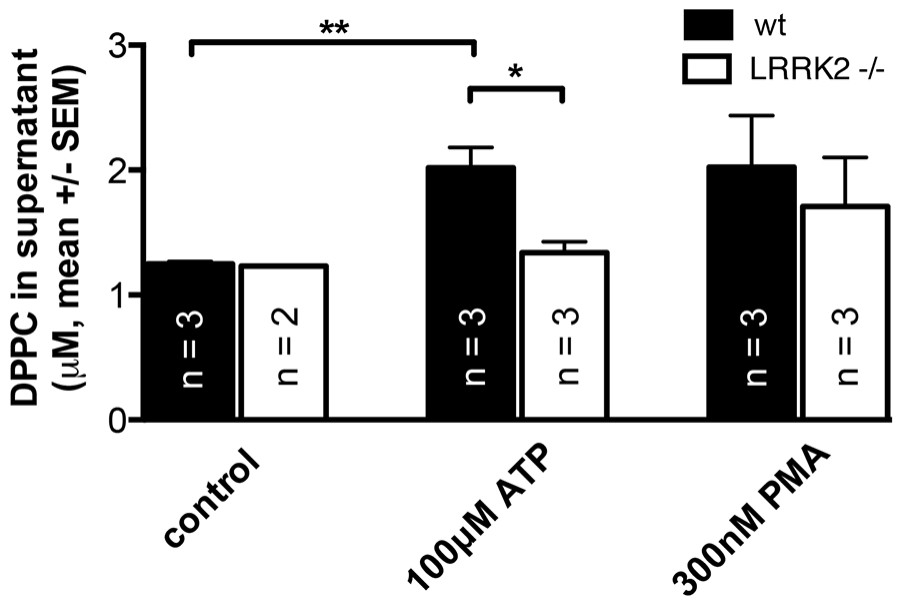
DPPC concentration in supernatant as a measure of surfactant secretion. DPPC concentration was measured in supernatant of unstimulated cells and of cells stimulated with either 100 µM ATP or 300 nM PMA for 30 minutes. DPPC concentration in supernatant was significantly higher in wt cells following stimulation with 100 µM ATP.

Based on our finding that knock-out of LRRK2 results in a significantly enhanced LB fusion response in ATII cells when stimulated with 100 µM ATP, we next examined whether this also results in increased surfactant secretion. We analyzed the amount of secreted phospholipids (DPPC is the main component of surfactant) using a recently described enzymatic protocol [53]. Surprisingly, 30 min after stimulation with ATP the phospholipid content of supernatants from LRRK2 -/- cells was significantly reduced compared to wt cells (1.34±0.09 µM, n = 3 for LRRK2 -/- cells vs 2.02±0.16 µM, n = 3 for wt cells, p = 0.02). The mean phospholipid content of supernatant was also decreased in LRRK2 cell cultures following stimulation with PMA, however the difference was not significant (1.7±0.39 µM, n = 3 for LRRK2 -/- cells vs 2.02±0.41 µM, n = 3 in wt cells, p = 0.6) (Figure 5).

### Actin coating

**Figure 6:**
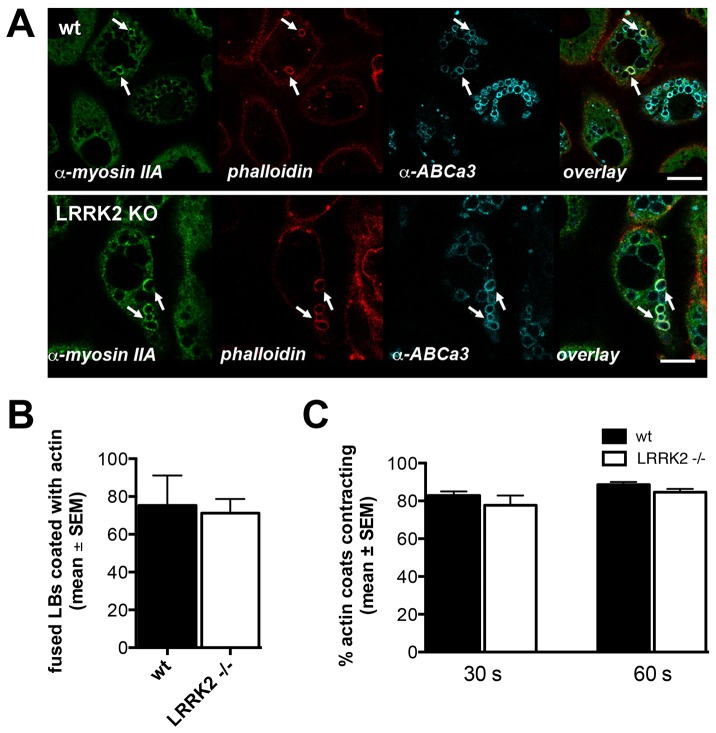
Actin coating and compression of fused LBs are not altered in LRRK2 -/- cells. A) Immunostaining of ATII cells from wt (upper image series) and LRRK2 -/- (lower image series) animals, labeled for myosin IIA, filamentous actin (phalloidin) and LB membrane marker ABCa3. Cells were fixed 3 minutes after stimulation with 100 µM ATP. Myosin IIA and phalloidin staining of ABCa3 positive organelles indicate actin coating of LBs following exocytic fusion with plasma membrane (arrows). Scale bar  = 10 µm. B) Percentage of LBs coated with actin-GFP following fusion after 100 µM ATP stimulation in live-cell experiments and C) fraction of actin coats that contracted within 30 s and 60 s following fusion. Data were derived from 2 wt and 2 LRRK2 -/- animals, and up to 67 fusions and 46 compression events were analysed for each animal. No significant difference was observed between wt and LRRK2 -/- cells.

To further elucidate these conflicting results – increased number of responding cells but reduced surfactant secretion in LRRK2 -/- cells – we investigated the potential impact of altered LRRK2 expression on the regulation of surfactant secretion during the post-fusion phase of LB exocytosis. The lipophilic nature of surfactant impedes rapid dispersal in aqueous solution and therefore surfactant does not readily diffuse out of fused LBs following opening of the exocytic fusion pore. We already showed that actin coating of fused LBs and subsequent actin coat compression play a pivotal role in surfactant expulsion from the vesicles [40], [41].Actin compression is assisted by non-muscle myosin II [40]. LRRK2 has already been demonstrated to be involved in actin remodeling in primary neurons by phosphorylation of ezrin-radixin-moesin family of actin-binding proteins [18]. Hence, we investigated a possible role for LRRK2 in actin coating of fused LBs. Immunocytochemistry revealed that, similarly to wt cells, actin and myosin II were recruited to fused LBs in LRRK2 -/- cells (Figure 6A). Detailed analysis of actin coat formation and compression in live-cell imaging experiments revealed no difference in the incidence of actin coating in cells transfected with actin-GFP (Figure 6B & C). Moreover, the kinetics of actin coat compression on fused vesicles after 30 s and 60 s in LRRK2 -/- cells was not different from wt cells.

## Discussion

Although LRRK2 was suggested to play a role in variety of diseases its cellular role remains elusive. Recent evidence implicated LRRK2 in regulation of secretory vesicle trafficking [20]–[22], [54] and in lysosomal pathways [26]–[29], [55]. In particular, one study found that genetic ablation of LRRK2 results in an increased number and size of secondary lysosomes in kidney proximal tubules cells and LBs in ATII cells in the lung [29]. It was the aim of this study to investigate whether the morphological changes observed in LBs from LRRK2 -/- animals have functional implications for LB exocytosis and surfactant secretion.

The original observation on increased LB size was made in LRRK2 -/- mice [29]. In this study we used LRRK2 -/- rats for functional studies, because rat, but not mouse ATII cells resemble a human phenotype [56] and all assays to study LB exocytosis and surfactant secretion are well established for primary ATII cells from rat [37], [43]. The results of our study showed that LBs in primary ATII cells isolated from LRRK2 -/- rats were significantly enlarged compared to cells from wt animals. This is in line with the initial observation in mice.

Stimulation of primary ATII cells with 100 µM ATP resulted in a significantly increased percentage of cells that responded to stimulation with exocytic activity when LRRK2 expression was ablated. LB exocytosis is exceptionally sensitive to [Ca^2+^]_c_ [34]. Ca^2+^ was described as a final trigger for LB fusion in ATII cells, the threshold necessary for fusion in ATII cells is very low (∼320 nM), and fusion kinetics correlate with [Ca^2+^]_c_ kinetics [42], [49]. The observation that resting [Ca^2+^]_c_ is not significantly elevated in LRRK2 -/- cells suggests that the increased rise in [Ca^2+^]_c_ upon stimulation with ATP is the main effector of the increased fusion response in these cells. It is easily conceivable that in LRRK2 -/- cells the threshold for LB fusion is reached more readily and exceeded for prolonged times and hence more cells exhibit LB fusions upon stimulation with ATP. Such a model is also supported by the observation that in LRRK2 -/- cells the exocytic response following ATP treatment is similar to the response following stimulation that causes maximum elevation of [Ca^2+^]_c_ and activation of Ca^2+^-dependent fusion activity (ionomycin). The increase in the rise of [Ca^2+^]_c_ upon ATP treatment in LRRK2 -/- cells could be due to altered Ca^2+^ levels in intracellular Ca^2+^ stores or increased Ca^2+^ release kinetics. It is unlikely to result from altered Ca^2+^ extrusion mechanisms as the time-course of the [Ca^2+^]_c_ decay is unchanged. Effects of LRRK2 on Ca^2+^ homeostasis and Ca^2+^ signaling have already been reported in other cell types [55], [57], [58]. In these systems overexpression of LRRK2 led to an imbalance in Ca^2+^ homeostasis [57] and profound Ca^2+^ release from lysosomal Ca^2+^ stores [58]. This is in contrast to the results within this study where ablation of LRRK2 enhances Ca^2+^ release from intracellular stores. This difference could be due to the different signaling mechanism underlying Ca^2+^ mobilization in the different systems. In ATII cells ATP elicits Ca^2+^ release via activation of IP_3_ receptors [42] whereas observations in HEK cells suggested a role for two pore channels (TPCs) in LRRK2 –mediated effects on Ca^2+^ mobilization [58]. The observation that the number of fusing LBs in responding cells was not significantly increased in LRRK2 -/- animals suggests that the observed effects of LRRK2 -/- act primarily at final stages in the LB fusion process. ATII cells lack a pool of “readily releasable” or “primed” LBs [31], [42]. Rather, LBs are constantly transported towards the plasma membrane and once in close proximity they fuse with the plasma membrane upon an appropriate stimulus [42]. The increased Ca^2+^ release in LRRK2 -/- cells following stimulation with ATP likely facilitates or accelerates fusion of LBs that are already localized close to the plasma membrane [42]. This is in line with the “triggered” fusion response observed in LRRK2 -/- cells where the majority of fusions occurred immediately after stimulation. However, due to the transient nature of the rise in [Ca^2+^]_c_ it barely affects recruitment or trafficking of additional LBs to the plasma membrane for fusion (only a slight increase in the number of fusions in individual responding cells was observed).

LRRK2 -/- does not seem to affect Ca^2+^ independent LB fusion activity as PMA induced LB fusion response is unchanged in LRRK2 -/- cells. However, we cannot fully exclude the possibility that PMA already causes a maximum stimulatory effect that cannot be increased upon. It is well established that PMA causes a strong fusion response (i.e. more cells responding within a defined period of time after stimulation) due to the long lasting stimulatory effect. Hence the combined application of ATP and PMA, evoking a Ca^2+^ increase as well as direct stimulation of protein kinase C, does not necessarily need to exceed the PMA response [42].

The observed increase in exocytic activity in LRRK2 -/- cells is also in line with observations from cortical neurons where LRRK2 silencing increased fusion kinetics [21] and with observation that gain-of-function mutation in LRRK2 results in impaired catecholamine and dopamine secretion [20], [54]. However, whether changes in exocytic activity in these and other cells are also linked to changes in Ca^2+^ signaling remains to be answered.

Shin et al showed that LRRK2 silencing in neurons leads to impaired compensatory endocytosis [22]. We did not observe this effect in ATII cells; however this could be due to the fact that endocytosis is rarely observed in cultured ATII cells [59].

Although ATII cells from LRKK2 -/- rats contain larger LBs and these LBs had an increased propensity for fusion with plasma membrane upon physiological stimulation, the amount of secreted phospholipids following stimulation was lower than in wt cells. Therefore, we tested the possibility that the extrusion of secretory material might be impaired in LRKK2 -/- cells. Surfactant is a poorly soluble substance and the opening of the exocytic fusion pore is not sufficient for efficient surfactant release from the fused vesicle. Actin coating of fused LBs and myosin driven compression of this coat is necessary for active surfactant extrusion [40], [41]. In addition, LRRK2 has been reported to influence actin remodeling [18] so we analysed actin coat formation and compression in ATII cells from LRRK2 -/- rats by immunostaining and in live-cell experiments. These experiments revealed no significant differences in actin coat formation and compression between LRRK2 -/- and control cells. It is hence unlikely that impaired actin coat formation and vesicle compression account for decreased phospholipid extrusion in LRRK2 -/- cells. Another possible explanation for the observed decrease in phospholipid secretion could be an impaired packaging of surfactant in LBs of LRRK2 -/- cells or decreased phospholipid content in LRRK2 -/- surfactant. Although the findings presented in this study (expression and localization of LB membrane markers and specific surfactant proteins, loading of LBs with fluorescent phospholipids, staining with Nile red, LB luminal pH) indicate that basic characteristics of LBs are not significantly altered in ATII cells from LRRK2 -/- rats we cannot exclude that lipid loading/packing of LBs accounts for the observed decrease in DPPC secretion. Hence further high-resolution studies using biochemical as well as ultra-structural techniques will be required to fully investigate surfactant composition in LBs from wt and LRRK2 -/- rats.

In summary our results suggest that LRRK2 -/- affects LB size, modulates intracellular Ca^2+^ signaling and promotes LB exocytosis upon stimulation of ATII cells with ATP. However, further studies are required to fully elucidate the molecular mechanism how LRRK2 regulates intracellular Ca^2+^ release in these cells and whether LRRK2 affects surfactant loading of LBs. Results of these studies will also shed some more light on whether the observed effects of LRRK2 on exocytosis and secretion in other cell types are linked to changes in intracellular Ca^2+^ signaling.
